# Proceedings of the Louisville Medical Club

**Published:** 1855-09

**Authors:** John Bartlett

**Affiliations:** Secretary


### ART. II.—PROCEEDINGS OF THE LOUISVILLE MEDICAL CLUB.

REPORTED BY JOHN BARTLETT, M. D., SECRETARY.

THE Louisville Medical Club met at the house of Dr. Bemiss on Tuesday evening, September 4th. President Ewing in the chair, and the following gentlemen present: Drs. Gross, Knight, Bemiss, Wible, Palmer, Rogers, Raphael, D. W. Yandell, Hardin, Bartlett, and Thompson.

Dr. Taylor, of Caseyville, Ky., was introduced to the Club by Dr. D. W. Yandell.

The paper of the evening was read by Dr. Knight on the

### USE AND EFFECTS OF CHLOROFORM IN LABOR.

*Gentlemen of the Louisville Medical Club:*—I am aware that there exists at the present time a great diversity of opinion among medical men as to the propriety of the use of chloroform in obstetrical practice, and while many of the most distinguished names of the profession warmly advocate its use, others equally distinguished, and probably as numerous, use it either with extreme caution or condemn it altogether.

I shall endeavor in the few remarks that I propose making, to confine myself to a plain and simple statement of the facts as they have presented themselves to me relative to the use and effects of chloroform in parturition.

It is almost needless to say that the chief agencies which effect the delivery of the child from the cavity of the womb are powerful uterine contractions, assisted in some degree by the diaphragm and abdominal muscles. It is by these that the expulsion of the child is effected, and anything that has a tendency to impair or paralyze these agencies or efforts of nature, has a corresponding tendency to prolong the period of labor.

No agent yet discovered possesses a more perfect and complete control over muscular action than chloroform. Blood-letting, opium, tobacco, and antimony, each powerful in effect, and, before the application of chloroform, the chief means of effecting muscular relaxation, are now regarded as comparatively feeble, and, in many cases, I fear, are wrongfully superceded by chloroform.

From an experience of several years in the use of chloroform in obstetrical practice, I am compelled to acknowledge that I have been entirely disappointed in the estimate that, from the representations of gentlemen of acknowledged worth and ability in the profession, I was at first led to place upon it. The most objectionable feature that is presented to my mind in the use of this agent in labor is, that it produces a weak and inefficient action of the uterus, and thereby paralyzes the most powerful and important agent concerned in the expulsion of the child.

Not only reason but experience proves this (but too clearly) to be the result of its action. For while we admit the effect of its power upon the muscular system generally, we are at the same time compelled to admit a similar action upon the muscles of the uterus, and whatever amount of muscular relaxation and debility may be induced by means of the agent employed, precisely in that ratio will the expulsive efforts of the uterus be rendered inefficient or null.

In a very large majority of cases in which I have used chloroform, the conviction of its tendency to greatly prolong the natural period of labor has been forced upon me, and in several instances where every circumstance seemed to indicate a speedy and easy termination of this natural process, in which I was persuaded by the patient or her friends to use chloroform, the expulsive efforts of the uterus were so enfeebled as to render that which, as I have remarked, at first gave every evidence of an easy and rapid termination, both a slow, tedious, and difficult process.

In face and breech presentations, where the life of the child is always endangered in proportion to the length of the labor, I would on no account advise the use of chloroform, and I think the same remark applicable to any other case of labor where a speedy termination from any cause is desirable.

Although I have been greatly disappointed in my expectations as to the value of chloroform in obstetrical practice, I would not be understood as condemning its use. That there are cases in which it is not only admissible but extremely useful I am perfectly willing to admit, and at the same time that I would censure a too general or exclusive resort to its use, I would not banish it altogether from the list of useful agents employed in obstetrical practice.

I am perfectly satisfied that in two cases which recently came under my observation, great benefit resulted from its employment. These were both cases of arm presentation, and in each the liquor amnii had been evacuated for several hours; there was perfect contraction, and complete rigidity of the os uteri, and after having used the ordinary means for the promotion of dilatation, without rendering the parts sufficiently lax to introduce my hand, I resorted to chloroform. As soon as the effect of the chloroform was sufficiently manifest I made an examination, and to my delight I found in each case complete relaxation of the previously rigid structures, introduced my hand without difficulty and was able to effect turning with great ease. Now, in cases of this description—although I admit they are rare—I think chloroform not only admirably adapted but imperatively called for. Indeed, in most cases of excessive uterine contraction, and in all in which rupture of the uterus is threatened, I should think chloroform was indicated unless there was some special constitutional objection to its use.

My experience with anæsthetic agents in operative midwifery is not very large, and so far as it goes has not inclined me to form a favorable opinion of their use. I do not know in how many cases requiring an operative procedure I have administered chloroform, but I am very positive that I have derived no benefit from its use in a single instance; and in some cases where the forceps were required, as well as in a few cases of craniotomy,

I am sure that it proved injurious by depriving the patient of consciousness, which all practitioners are aware is an indispensable safeguard.

It will be observed from the preceding remarks that the range which I concede to chloroform in midwifery is very limited. If I were called on to express in a single line the only circumstances in which my experience leads me to think anæsthetics either demanded or expedient in parturition, I should say *they are required only in powerful*, *or irregular*, *or excessive uterine action.* And if I add to this, spasmodic retraction and rigidity of the neck, nervous excitement and intellectual disturbance, I have included the whole catalogue of conditions in which I am now ever disposed to insist upon the induction of anæsthesia.

I claim to have awarded to this agent a fair and impartial trial in obstetric practice, and believe I am wholly free from any further prejudice against its use than has naturally and I hope logically grown out of a somewhat extensive experience in its application. No one would hail with greater pleasure than myself the discovery of an agent which, while it would annul the pains of childbirth, would neither prolong the process nor endanger the life of the patient. Chloroform, however, I am free to say, has not in my hands proved to be this agent, and I cannot but condemn the use which is now so generally made of this most potent and oftentimes hazardous remedy for the single purpose of allaying pains which, as every one knows, so seldom in themselves prove fatal.

I but repeat a truism when I remark that in the great majority of cases of natural labor, if the pains be regular, women are exposed to but little danger; and I belong to that school of practitioners who hold that the less manual or medicinal interference with a process which will terminate spontaneously and safely the better.

I am aware that my views upon this subject are neither the most general nor popular among parturient women, whose fears, it is well known, are easily excited, and who are ever ready to try any agent which promises even a temporary suspension of their sufferings. I am also aware that among females in this condition the obstetrician who promises the greatest immunity from pain is most in request. But I do not wish to be considered as implying in the above remark any suspicion of the honesty or candor of those who differ with me in opinion.

To conclude the brief, and I feel very immethodical remarks I have presented to the Club, I wish to reiterate the following summary of my experience:

1st. Chloroform, administered in parturition, produces great muscular relaxation and thereby renders the expulsive efforts of the uterus feeble and inefficient.

2d. It causes partial if not total suspension of voluntary muscular effort, which tends greatly to prolong the natural period of labor, and thereby invites the attendant dangers of this condition.

3d. It is inadmissible in uterine hemorrhage.

4th. It is inadmissible in operative midwifery.

5th. It is inadmissible in all presentations in which the life of the child is endangered in proportion to the prolongation of the labor.

Dr. Gross inquired in what way and to what extent Dr. Knight gave chloroform?—to which he replied, he did not give it as surgeons do, to entire unconsciousness, but simply to blunt sensibility—to annul to some extent the pains. He had administered it in all stages of labor, before free dilatation of the os in some cases, though he had generally confined himself to the latter stages. The patients inhaled the choloroform either from a sponge or pocket handkerchiet.

Dr. Hardin dissented from many of the views expressed by Dr. Knight; and while he was willing to admit that complete anæsthesia would perhaps retard and in some instances altogether arrest uterine action, yet he thought it was every day carried sufficiently far to afford great relief to parturient women without in any degree lengthening the period of their labors. Coinciding Dr. K.'s position, that anæsthetics weakened the force of the pains, he thought this more than compensated by the relaxation of the perineum which almost always followed their use. He had not seen a case of serious uterine hemorrhage in which chloroform was administered. He thought anæsthesia advisable in operative midwifery, and regarded it as almost a *sine qua non* in convulsions. He remembered a case that he was once called to see in which convulsions, alternating with coma, had been present for twelve hours, which, under chloroform, was promptly relieved. When the chloroform was first exhibited the uterus had hardly begun to dilate, yet in about five hours the child was delivered. He could not avoid believing that without an anæsthetic this patient would have succumbed. He has not remarked any greater mortality among puerperal women in his practice since the introduction of chloroform than before, and he has delivered no still-born children.

Dr. Gross thought the action of the uterus was not weakened if the inhalation of chloroform was stopped short of full anæsthesia. He had been particularly pleased with the effect of chloroform in cases where there was great irritability of the uterus with frequent and inefficient and harassing pains. He had administered chloroform to women who, in previous confinements, had suffered from uterine hemorrhage, without the occurrence of any sanguineous losses. No still-born children had occurred in his practice as the result of anæsthesia of the mother.

Dr. Wible regarded the effect of anæsthetics as certainly retarding labor, and thought there could be but little doubt of their increasing the tendency to hemorrhage.

Dr. Caldwell is not in the habit of proposing chloroform to his patients, but gives it when it is asked for. He is confident he has several times seen labor completely suspended by its use. He has never known hemorrhage to follow its administration, and when the hemorrhage depends on irregular contractions so far from chloroform being contra-indicated he thought it specially indicated.

Dr. Bemiss said that he had not given chloroform in a very large number of midwifery cases. In one instance he is sure that labor was retarded by it.

Dr. Thomson is not in the habit of using chloroform for the production of full anæsthesia. He gives it cautiously for the mitigation of pain.

Dr. D. W. Yandell had administered chloroform in only nineteen cases. He had never known any unpleasant consequences from it, and agreed fully with the views expressed by Dr. Hardin.

Dr. Ewing seldom prescribes it, and thinks that whenever he has used it to any great extent that the labors have been prolonged. No children were still-born in his practice.

Dr. Rogers had used chloroform repeatedly, though always for alleviation of the pains rather than with the view of totally destroying sensibility. It was no longer a question with him in regard to the influence that chloroform exerted upon uterine contractions. He was satisfied that it retarded them, and he would remark that this opinion was based upon a considerable number of carefully conducted experiments. He could refer to not a few instances of labor which were managed at one time with and at another time without chloroform, and he was sure that those were most tedious in which the anæsthetic was exhibited. He thought it disposed to flooding.

Dr. Thomson's report on the use of chloroform in surgery was deferred till the next meeting of the Club.

### MELANOSIS AND SCIRRHUS.

Dr. Gross exhibited a jar of specimens of black and white cancer, which he removed from the body of a gentleman, aged fifty-eight, who died under his charge on the 22d of August. He had been in attendance for the last eight months on the patient, on account of carcinoma of the anus and rectum, attended with the development of numerous black tubercles beneath the skin. The disease was first noticed about twelve months previously to the death of the patient, who died in a state of gradual exhaustion. The prominent symptoms were pain and soreness of the anus, with occasional hemorrhage from the rectum, frequent diarrhœa, and progressive emaciation. The appetite was generally good, and there was no reason to believe, from anything present, that any of the other organs of the body were seriously affected, as was found to be the case on the dissection. During the early stage of the attack the bladder was very irritable, and the frequent use of anodyne suppositories was required to afford relief.

The dissection revealed the existence of black and white cancer in almost every organ of the body—in the skin and subcutaneous cellular substance, the lymphatic ganglions of the groin,

---

NOTE.—It is the intention of Dr. Gross to give a detailed and circumstantial account of this extraordinary case in some future number of this Journal.

the stomach, small and large bowel, the liver and gall-bladder, pancreas, the omentum and peritoneum, the kidneys and supra-renal capsules, the bladder and prostate gland, the lymphatic ganglions of the abdomen, the lungs, pleura, heart, bronchial ganglions, and the thyroid gland. The brain was not inspected. The œsophagus, spleen and large vessels were free from disease. Thousands of tubercles, from the size of a pin's head to that of a split pea, existed in the subperitoneal cellular tissue. They were, for the most part, of a rounded shape, very hard, and of a black-ish color at the periphery with a white central nucleus. The supra-renal capsules and the lymphatic ganglions of the abdomen consisted almost entirely of black matter. The former of these bodies was enormously enlarged. The heart presented numerous tubercles, of a light, slate color, and from the volume of a hemp-seed to that of a hazle-nut. They were most conspicuous in the right auricle and ventricle, especially the former. The bladder contained five small characteristic tumors, and the prostate gland was of a dense scirrhous consistence and charged with an abundance of black matter.

Dr. Gross then proceeded to exhibit an interesting example of

SCIRRHUS OF THE STOMACH.

The following is an abstract of the case:

W. L. S., aged forty-eight, a planter of Louisiana, the father of six children, and a man of temperate habits, was attacked with symptoms of dyspepsia in the summer of 1850, his health having up to that time been tolerably good, although rather delicate. Gradually nausea and vomiting ensued, attended with a sense of fullness and uneasiness in the epigastric region. The appetite was generally indifferent, and there was frequent annoyance from acidity of the stomach and eructations of gas. Occasionally there were griping pains, with now and then a pretty severe attack of colic. The bowels were generally inclined to constipation. There was gradual failure of strength, with progressive emaciation, and the countenance wore a sallow and dejected appearance. During the six months immediately preceding his dissolution, Mr. S. suffered almost constantly from nausea, and had frequent vomiting, consisting, for the most part, of thick, ropy, brownish mucus, mixed with ingesta. At first the vomiting seldom occurred under two, three or four hours after eating; but latterly it often took place almost immediately, or, at all events, within in a very short time, after using food or drink. I saw the patient, for the first time, about the middle of August, along with Dr. Chenoweth. I found him very feeble, and much emaciated, with a dark, sallow countenance, almost constant nausea, and frequent vomiting. His appetite was gone, and the bowels were costive, and he complained of pain, of a dull, aching character, in the right hypochondriac and epigastric regions, which was increased by pressure and change of posture. But little was attempted in the way of treatment, it being apparent that the case must soon terminate fatally. He lingered on, becoming gradually more and more exhausted, till the 30th of August.

The only organ which was found to be diseased was the stomach, which was enormously distended, and nearly entirely closed at the pylorus. It contained, by estimate, upwards of half a gallon of fluid, of the appearance of weak coffee; the mucous coat was, throughout, unusually red and congested, and the muscular fibres were developed in an uncommon degree. The pylorus was the seat of scirrhous degeneration, involving its entire circumference, and encroaching so much upon the cavity of the tube as to leave an opening hardly large enough to receive the end of the little finger. The mucous membrane at and around the morbid mass was abnormally vascular and thickened, but not ulcerated.

Dr. Rogers mentioned a case of

### NECROSIS OF THE FEMUR, with the following history: The patient, a man, aged thirty, has had necrosis of the thigh-bone since his eighth year. It began without external injury, simply as the result of inflammation, and has discharged itself through six or seven sinuous outlets without intermission ever since. Twenty-two years after the disease set in a thin shell of bone, about two inches long, was detached from the femur and came away. A short time ago a second fragment was removed. The condition of the patient at this time is about as follows: General health good; pain of affected limb trifling; the knee-joint is nearly stiff, and the limb slightly curved, and considerably shortened—about a drachm of matter is discharged daily from the openings above the knee-joint.

